# Ex vivo ^1^H nuclear magnetic resonance spectroscopy reveals systematic alterations in cerebral metabolites as the key pathogenetic mechanism of bilirubin encephalopathy

**DOI:** 10.1186/s13041-014-0087-5

**Published:** 2014-11-26

**Authors:** Wenyi Hu, Xiaojie Cheng, Xinjian Ye, Liangcai Zhao, Yanan Huang, Huanle Zhu, Zhihan Yan, Xuebao Wang, Xiaojie Wang, Guanghui Bai, Hongchang Gao

**Affiliations:** Radiology Department of the Second Affiliated Hospital, Wenzhou Medical University, Wenzhou, 325027 Zhejiang China; Institute of Metabonomics & Medical NMR, School of Pharmaceutical Sciences, Wenzhou Medical University, Wenzhou, 325035 Zhejiang China

**Keywords:** Bilirubin encephalopathy, Nuclear magnetic resonance spectroscopy, Metabonomics, Gln-Glu-GABA cycle

## Abstract

**Background:**

Bilirubin encephalopathy (BE) is a severe neurologic sequelae induced by hyperbilirubinemia in newborns. However, the pathogenetic mechanisms underlying the clinical syndromes of BE remain ambiguous. *Ex vivo*^1^H nuclear magnetic resonance (NMR) spectroscopy was used to measure changes in the concentrations of cerebral metabolites in various brain areas of newborn 9-day-old rats subjected to bilirubin to explore the related mechanisms of BE.

**Results:**

When measured 0.5 hr after injection of bilirubin, levels of the amino acid neurotransmitters glutamate (Glu), glutamine (Gln), and γ-aminobutyric acid (GABA) in hippocampus and occipital cortex significantly decreased, by contrast, levels of aspartate (Asp) considerably increased. In the cerebellum, Glu and Gln levels significantly decreased, while GABA, and Asp levels showed no significant differences. In BE 24 hr rats, all of the metabolic changes observed returned to normal in the hippocampus and occipital cortex; however, levels of Glu, Gln, GABA, and glycine significantly increased in the cerebellum.

**Conclusions:**

These metabolic changes for the neurotransmitters are mostly likely the result of a shift in the steady-state equilibrium of the Gln-Glu-GABA metabolic cycle between astrocytes and neurons, in a region-specific manner. Changes in energy metabolism and the tricarboxylic acid cycle may also be involved in the pathogenesis of BE.

## Introduction

Neonatal hyperbilirubinemia, which is caused by immaturity of hepatic conjugation and clearance processes for unconjugated bilirubin (UCB) [1], is a common and important pathological condition that occurs in approximately 60% of all term newborns and 80% of all pre-term infants [2]. The outcome for the majority of babies is benign, but untreated neonates or newborns with very high UCB levels can develop yellow staining and the neurological dysfunction characterizing bilirubin encephalopathy (BE). A substantial number of research studies have recently demonstrated that survivors of BE always exhibit a sequence of severe neurological sequelae, including choreoathetosis, gaze paresis, hearing loss, and, more rarely, developmental delays [3]. All of these pathological conditions present an important threat to infant health and place significant burdens on neonates.

The mechanisms underlying BE neurotoxicity are unclear but generally accepted to be related to the deposition of UCB in the central nervous system (CNS) [4]. UCB, which is produced from the degradation of heme, serves as an antioxidant and cytoprotective agent at physiological levels; however, mildly elevated concentrations may inhibit neuronal functions and affect viability [5]. Relevant studies on UCB have attempted to determine its neurotoxicity based on the incubation of neural cells, such as astrocytes and neurons isolated from rat cortical cerebrums with UCB [6-8]. UCB neurotoxicity may be associated with the accumulation of reactive oxygen species, breakdown of glutathione redox status, dysfunction of the mitochondria, and even cell death [7,8]. Researchers have also indicated that UCB induces enhanced oxidative stress, alterations in neurogenesis, neuritogenesis, synaptogenesis, and disruption of neuronal network dynamics [9]. However, which of these mechanisms is most important in the causation of the clinical syndromes of BE has yet to be investigated.

Over the last decade, the rapidly growing research field of metabolic changes in neurotransmitters has introduced new insights into the pathology of neurodegenerative disorders as well as methods to predict disease onset. For example, Sanacora studied the concentrations of γ-aminobutyric acid (GABA) and glutamate (Glu) in major depression patients using magnetic resonance spectroscopy and found that decreased levels of occipital cortex GABA and increased levels of Glu may serve as a biological marker for a subtype of major depressive disorder [10]. Kahn observed altered Glu, aspartate (Asp), glycine (Gly), and GABA concentrations in the striatum of ischemia rats by high performance liquid chromatography and suggested that neurotransmitter release following cerebral ischemia may serve as a potential mechanism of ischemia [11]. Campos found cerebral metabolic disorders in a rat model of stroke [12], as well as high levels of Glu in brain stroke patients [13]. Therefore, studying metabolic changes in cerebral metabolites at the molecular level could lead to elucidation of a possible link between novel mechanisms of BE and therapeutic interventions for the disease.

^1^H NMR has been used previously to investigate, in vivo and ex vivo, cerebral metabolic changes in the central nervous system of both patients and animals. Ex vivo ^1^H NMR is usually performed at higher field strength, which gives higher detection sensitivity, improved dispersion of metabolite peaks, and better regional specificity. Using this approach, we have recently studied the cerebral metabolites in Parkinson’s disease rats and shown that the metabolic characteristics in the left and right striatum are region-specific [14]. In the present study, changes in the concentrations of cerebral metabolites in the hippocampus, occipital lobe, and cerebellum of newborn 9-day-old rats subjected to bilirubin were measured using ex vivo ^1^H NMR. Significant changes in the levels of neurochemicals were found; these changes may provide novel clues for elucidating the mechanisms underlying BE.

## Results

### Serum and brain bilirubin concentrations and histopathology

Compared with the control group, the serum levels of bilirubin in the BE group were markedly elevated 0.5 hr after injection (531.54 ± 27.58 μmol/L vs 5.39 ± 1.54 μmol/L, p < 0.001). With the passage of time, bilirubin concentrations gradually decreased and reached to 11.26 ± 3.15 μmol/L in BE 24 hr rats. By contrast, no significant differences were observed among the control groups at different time points. Bilirubin was not detected in the brain of control rats, but bilirubin levels in BE 0.5, 4, and 24 hr rats were 4.68 ± 0.35, 8.61 ± 0.53, and 6.89 ± 1.25 nmol/g brain tissue weight, respectively.

**Figure 1:**
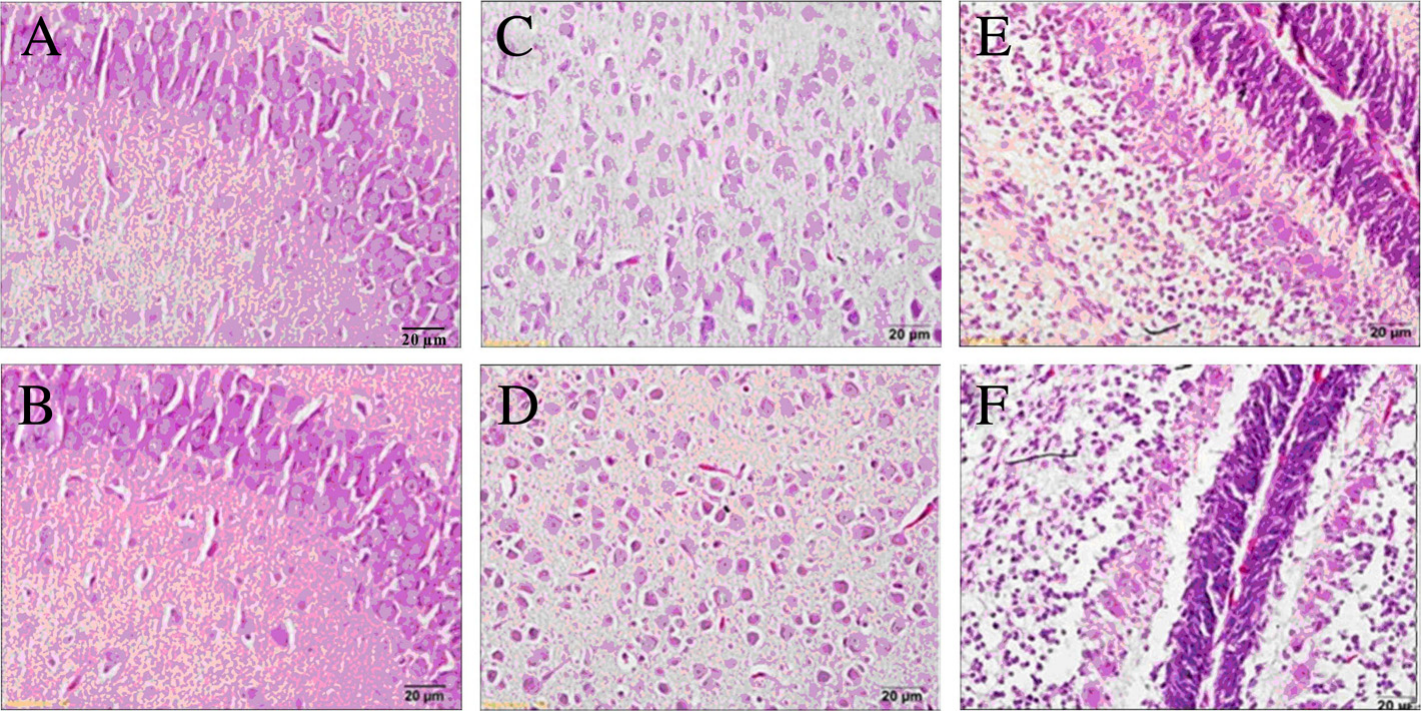
**Representative Mayer’s hematoxylin- and eosin-stained sections (400 fold) of hippocampus, occipital cortex and cerebellum from control (A, C, E) and BE 4 hr rats (B, D, F)**.

Microscopic examination showed that representative Mayer’s hematoxylin-eosin- stained sections of the three brain regions from control and BE 4 hr rats (Figure 1). Regardless of the brain tissues tested, cells of controls maintained a normal morphology and were arranged in neat rows without any inflammatory reaction (Figure 1A, C, E). By contrast, BE rats showed significant histological abnormalities with sparse organization structures, disorganized swollen neurons, and changes in vacuolation in all three brain regions, particularly the hippocampus (Figure 1B). These histopathological results confirm the formation of apparent lesions and functional changes in the brain tissues of BE 4 hr rats, also the damage in the hippocampus last for long periods of time.

### NMR spectra and multivariate statistical analysis

**Figure 2:**
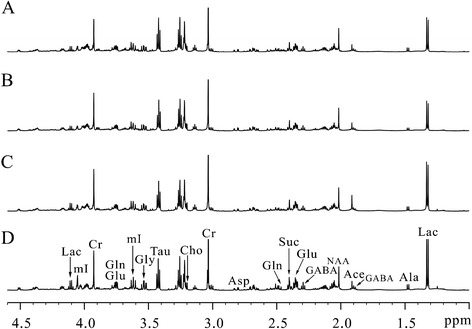
**Representative** ^**1**^ **H NMR spectra of the hippocampus extracts obtained from one control rat at 0.5-h (A) and three BE rats at 0.5 hr (B), 4 hr (C) and 24 hr (D).**

Representative ^1^H NMR spectra of the hippocampal extracts obtained from one control rat at 0.5 hr (A) and three BE rats at 0.5 hr (B), 4 hr (C), and 24 hr (D) are shown in Figure 2. Assignments presented in Figure 2A are based on our published work [15] and verified by 2D ^1^H–^1^H COSY and TOCSY spectra (data not shown). ^1^H NMR spectra of brain tissue extracts allow the simultaneous measurement of numbers of endogenous metabolites, such as lactate (Lac), alanine (Ala), N-acetyl aspartate (NAA), GABA, Glu, succinate (Suc), Gln, Asp, creatine (Cr), choline (Cho), taurine (Tau), Gly, and myo-inositol (mI).

**Figure 3:**
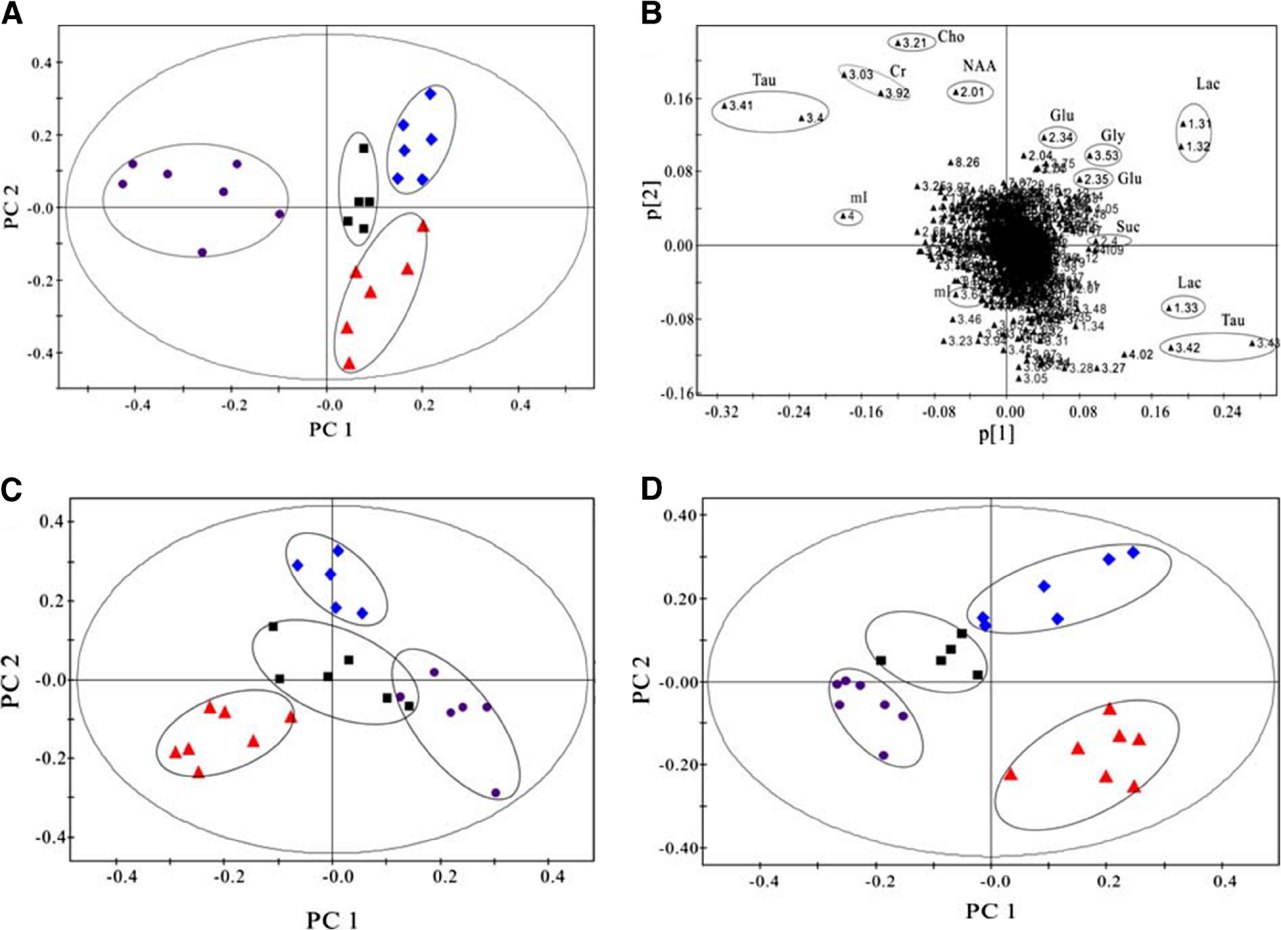
**PLS-DA scores plots of PC1 and PC2 in the hippocampus (A), occipital cortex (C), and cerebellum (D) of BE rats at 0.5 hr (diamond), 4 hr (triangle), 24 hr (circle) and controls (Black square), and the corresponding loading plot of hippocampus (B).**

Partial least squares-discriminant analysis was performed based on the hippocampal, occipital cortex, and cerebellar ^1^H NMR spectra of the BE 0.5, 4, and 24 hr rats as well as corresponding controls (Figure 3). As shown in the hippocampus (Figure 3A), BE groups are gradually away from the control group over time, especially BE 24 hr rats, which displayed clear discrimination along the direction of the first principal component. The corresponding loading plot (Figure 3B) for the first two principal components showed that Lac, Glu, Gln, mI, NAA, and Tau are among the major contributors to the separation. In the occipital cortex (Figure 3C), BE 0.5, 4, and 24 hr rats and corresponding controls separated from each other, and BE groups were scattered around the control group. For the cerebellum (Figure 3D), the BE 4 hr group displayed a distinct separation from the control group, and data on BE 24 hr rats were clustered closer to the controls.

### Metabolite concentrations in various brain regions

**Figure 4:**
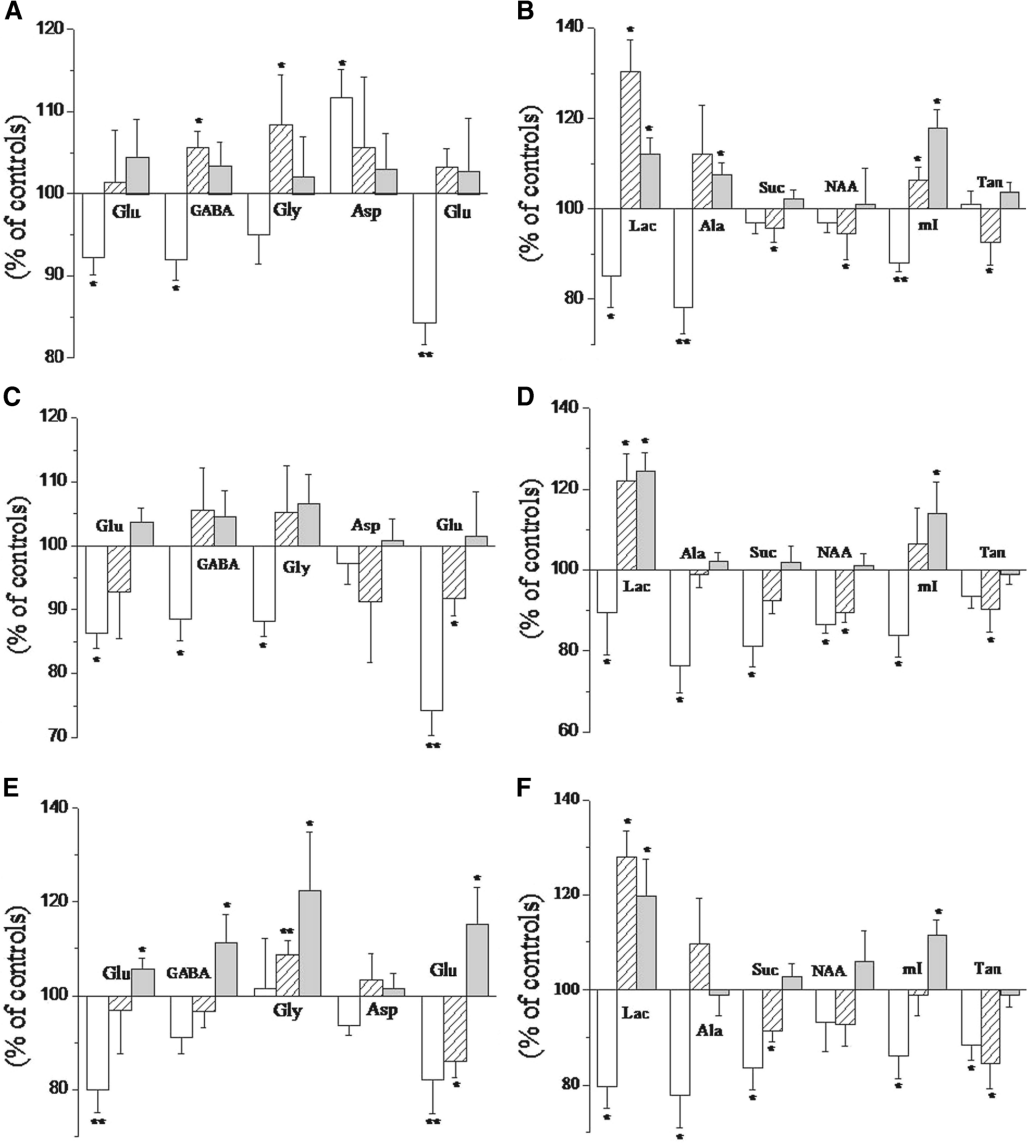
**Changes in the percentage metabolite concentrations (% of controls) in the hippocampus (A, B), occipital cortex (C, D), and cerebellum (E, F) at 0.5 hr (blank), 4 hr (slashes) and 24 hr (gray).** Significant differences were assigned as follows: single asterisk *p* < 0.05; double asterisks *p* < 0.01.

The concentrations of metabolites measured in the hippocampus, occipital cortex, and cerebellum of the control rats at 0.5 hr are listed in Table 1. Changes in metabolite concentrations at different time points relative to the control group (100%) are shown in Figure 4. In the hippocampus (Figure 4A and B), almost all of the amino acid neurotransmitters were significantly lower in BE 0.5 hr rats than in other groups (92.3 ± 2.15% for Glu, p < 0.05; 92 ± 2.5% for GABA, p < 0.05; and 84.3 ± 2.7% for Gln, p < 0.01), except for Asp, which showed markedly higher levels (111.7 ± 3.4%, p < 0.05). Changes in amino acid neurotransmitters were reversed in BE 4 hr rats, and all metabolic changes returned to the normal in BE 24 hr rats. In the occipital cortex (Figure 4C and D), nearly all of the amino acid neurotransmitters were reduced in BE 0.5 hr rats, similar to observations in the hippocampus. By contrast, concentration changes were not statistically significant in BE 4 and 24 hr rats, except for Gln, the levels of which remained low after 4 hrs. Differing from metabolic changes in the hippocampus and occipital cortex, Glu and Gln levels significantly decreased in the cerebellum (Figure 4E and F), whereas levels of GABA, Gly, and Asp showed no statistically significant differences in BE 0.5 hr rats. Metabolite concentrations then progressively increased. Gly concentrations reached statistical significance at 4 hrs, and almost all of the metabolites were significantly increased at 24 hrs.

**Table 1:** **Concentrations of cerebral metabolites (mmol/kg wet tissue weight) in the hippocampus, occipital cortex and cerebellum of control 0.5 hr rats measured by ex vivo** ^**1**^ **H NMR spectroscopy**

**Metabolites**	**Integral (ppm)**	**Hippocampus**	**Occipital cortex**	**Cerebellum**
Lac	δ1.31-1.34	7.73 ± 0.95	9.70 ± 2.18	5.37 ± 0.74
Suc	δ2.39-2.41	5.53 ± 0.51	7.73 ± 0.79	5.50 ± 0.24
Ala	δ1.46-1.49	1.39 ± 0.16	1.91 ± 0.30	1.09 ± 0.12
Glu	δ2.33-2.38	8.75 ± 0.71	11.89 ± 1.53	5.76 ± 0.42
GABA	δ2.27-2.31	3.30 ± 0.23	3.97 ± 0.55	1.96 ± 0.17
Gly	δ3.54-3.56	1.13 ± 0.09	1.54 ± 0.13	0.80 ± 0.14
Asp	δ2.80-2.82	1.95 ± 0.20	3.08 ± 0.21	1.30 ± 0.15
Gln	δ2.45-2.50	1.01 ± 0.08	1.37 ± 0.10	0.72 ± 0.13
NAA	δ2.01-2.02	3.24 ± 0.23	4.65 ± 0.53	2.05 ± 0.15
mI	δ3.60-3.64	4.65 ± 0.26	6.82 ± 0.84	5.17 ± 0.26
Tau	δ3.40-3.44	14.29 ± 1.11	21.84 ± 2.27	8.55 ± 0.49

The energy-related metabolites, Lac and Ala, manifested similar changes at different time points in the studied brain regions (Figure 4). In BE 0.5 hr rats, Lac and alanine levels markedly decreased; at 4 and 24 hrs, however, Lac increased whereas alanine showed no significant changes. Levels of succinate (Suc), an important tricarboxylic acid (TCA) cycle intermediate, decreased in the occipital cortex and cerebellum of BE 0.5 hr rats and subsequently returned to normal in the occipital cortex at 4 and 24 hrs. In the cerebellum, Suc levels remained low in BE 4 hr rats and eventually returned to normal at 24 hrs. Interestingly, no significant changes were observed in the hippocampus. NAA, which is commonly believed to be a neuronal marker, decreased by varying degrees in the studied brain regions of BE 0.5 and 4 hr rats; no significant changes were observed in BE 24 hr rats. mI, which is believed to be a marker of astrocytic activity, markedly decreased in BE 0.5 hr rats and significantly increased in BE 24 hr rats in all three brain regions; however, no significant changes were observed at 4 hrs. Another astrocytic activity marker, Tau, significantly decreased in the three studied brain regions of BE 4 hr rats and eventually returned to normal levels at 24 hrs.

## Discussion

NMR is a powerful approach for studying brain energy metabolism, neurotransmission, and glial–neuronal interactions [16,17], and it has been previously used to investigate the changes in brain metabolism of various encephalopathies [14,15,18]. In the present study, changes in the concentrations of cerebral metabolites in the hippocampus, occipital cortex, and cerebellum of rats subjected to BE 0.5, 4, and 24 hrs by ex vivo ^1^H NMR spectroscopy were comprehensively reported. Obvious metabolic perturbations of neurochemicals were observed, including some important excitatory and inhibitory neurotransmitters and energetic products, as well as a marker of neurons and astrocytic activity in various cerebral regions of BE rats. Bilirubin-induced changes in metabolite concentrations exhibited notable features at 0.5, 4, and 24 hrs, respectively. Furthermore, the disorders of neurochemical metabolism varied in the hippocampus, occipital cortex, and cerebellum of BE rats, suggesting specific impairment.

### Changes in amino acid neurotransmitter concentrations

Gln, Glu, and Asp are primary excitatory neurotransmitters whereas GABA and Gly are the main inhibitory neurotransmitters in the CNS [19]. Concentration changes in these amino acid neurotransmitters can reflect alterations in the balance between excitatory and inhibitory processes in the brain. In BE 4 hr rats, levels of Gly and GABA significantly increased in the hippocampus, and levels of Gln significantly decreased in the occipital cortex and cerebellum; no statistically significant changes in Glu levels were observed in any of the brain regions studied. The results obtained were consistent with previously reported findings, wherein rat cortical astrocytes showed high levels of Glu release after 4 hrs of incubation with UCB [20,21]. We hypothesize that this phenomenon could serve as a defensive strategy to protect neurons from excitotoxic injury or as an indicator of astrocyte damage, which directly affects the transformation of Glu into Gln. We believe that long-term abnormalities in glutamatergic and GABAergic activities and the associated adaptive metabolism can induce disorders in the steady-state equilibrium of the Gln-Glu cycle shuttling between astrocytes and neurons [22,23], which plays an essential role in neurodegenerative disorders.

### Energy metabolism and changes in other metabolic equilibria

Lac can be used as an energetic substrate by glycolytic processes [24] when neural energy demands transiently exceed the rate of oxidative metabolism. Bilirubin has been reported to inhibit glycolysis [25], mitochondrial respiration [26], and oxidative phosphorylation [27,28], as well as the activity of enzymes of the TCA cycle [29]. Thus, significant increases in Lac levels, which are commonly believed to serve as a marker of mitochondrial dysfunction in the hippocampus, occipital lobe, and cerebellum of BE 4 hr rats in the present study, are most likely caused by brain switching to consume glycolysis products and maintain energy homeostasis. The above hypothesis was further confirmed by a widespread decrease in levels of Suc, which is an important intermediate product of TCA cycle, thereby indicating the inhibition of TCA cycle. Metabolic changes in Lac and Suc suggest that altered energy metabolism with changes in the TCA cycle is involved in the pathological mechanism of BE.

Significantly reduced concentrations of Tau, a marker for astrocytic activity that has important functions in regulating intracellular osmolarity of the astrocytes [30], were found in the hippocampus, occipital lobe, and cerebellum of BE 4 hr rats. The above results are consistent with the fact that Tau production is one of the defense mechanisms against excitotoxic conditions in the brain, considering that Tau is both an osmoregulator and a neuromodulator [31]. Significant reductions in the levels of NAA, which is commonly considered a metabolic marker reflecting the functional status of neurons and axons in the brain [32,33], were observed in the hippocampus and occipital lobe of BE 4 hr rats. These observations parallel in vivo ^1^H MRS findings, which demonstrate significant decreases in NAA/Cho and NAA/Cr ratios in neonates with BE [34]. Considering that the Cr peak is relatively stable, it is often used as an internal reference for the calculation of other neurometabolic changes. Reductions in NAA indicated neuronal and axonal loss or dysfunction, as well as abnormal increases in gliosis in the basal ganglia. Interestingly, increased mI concentrations, which suggest glial hypertrophy or proliferation, were also found in all three brain regions of BE 24 hr rats.

### Notable features of metabolism at BE 0.5, 4, and 24-hr rats

The decline in metabolite concentrations recorded in BE 0.5 hr rats is correlated with the results of a previous study, which indicated that severe bilirubin intoxication could induce a widespread decrease in metabolites [35]. Indeed, when large amounts of bilirubin accumulate within the CNS, local cerebral metabolic rates for glucose show widespread decreases studied by quantitative autoradiographic [^14^C]2-deoxyglucose method [36]. Metabolic perturbations in the neurochemicals of BE 4 hr rats in all three brain regions studied may be a manifestation of brain injury; these findings are linked to reports that UCB induces serious oxidative and nitrosative stress at 4 hr, engenders synaptotoxicity, and elicits cytoskeleton changes [9,37]. Brain damage could be further proven by histopathology results in BE 4 hr rats. The majority of the metabolite concentrations tended to normalize in BE 24 hr rats, except for Lac and mI in the three brain regions studied, Ala in the hippocampus, and the neurotransmitters in the cerebellum. The results obtained are consistent with those reported in previous studies, which show that the adverse effects of bilirubin on the brain and neurons may be reversible [38-40]. Further studies must be carried out to determine whether or not the reversibility of neurons is only temporary.

### Selective vulnerability of rat brain regions to BE

The brain is largely under development during the first weeks of postnatal life, and enhanced plasticity and myelination create selective brain regional vulnerability [8]. Different levels of antioxidant proteins in brain regions may account for differences in vulnerability [41]. The pyramidal cell layer of the hippocampus is noted to be particularly vulnerable to hypoxic-ischemic injury [42].

To investigate whether or not different brain regions show distinct susceptibilities to BE, we studied hippocampal, occipital cortex, and cerebellar tissues isolated from newborn rats. Interestingly, metabolite changes in the three tissues provided us with a potential explanation for the regioselectivity of BE injury. Figure 4 shows that metabolic patterns in the hippocampus, occipital cortex, and cerebellum were inconsistent over time, since the hippocampus suffered more severe metabolic disorders than the other regions, as confirmed by hematoxylin + eosin staining. We therefore hypothesize that the hippocampus is a vulnerable brain region and that BE-induced neurotoxicity may have adverse outcomes on future learning, memory, and cognitive functions. The metabolism disorders in the cerebellum appeared to be longer, which presents new perspectives for potential therapeutic approaches.

In conclusion, the present study was designed to detect systematic alterations in multiple brain metabolites and explore the related mechanisms of BE. Region-specific changes were observed in the concentrations and metabolism of the neurochemicals Lac, NAA, Glu, Gln, GABA, as well as other cerebral metabolites in the newborn rat hippocampus, occipital cortex, and cerebellum. The results suggest that BE leads to multiple neurochemical changes in different brain regions that may involve the activity and transition of neurotransmitters and brain energy metabolism, as well as other changes in metabolic equilibrium. The results obtained shed light on the mechanisms underlying BE neurotoxicity and provide an important reference by which to better understand BE in the clinical setting. The observed metabolic changes are closely related to the pathological mechanisms of damage in different brain regions and could be part of the adaptive measures employed by the CNS in response to BE.

## Materials and methods

### Animals

Newborn Sprague–Dawley rats weighing 19–21 g (9 days age), whose mother were purchased from the SLAC Laboratory Animal Co. Ltd. Shanghai, China (14-16 days of pregnancy, 280 ± 15 g) and were kept in a specific pathogen free (SPF) colony of the Laboratory Animal Center of Wenzhou Medical University (Wenzhou, China) with regulated temperature and humidity and a 12:12-h light–dark cycle with lights on at 8:00 a.m. During the whole experimental process, rats were fed with standard rat chow and tap water and all of the newborn animals were cared by their own mother rats. All animal treatments were strictly in accordance with the National Institutes of Health Guide for the Care and Use of Laboratory Animals.

### Jugular vein injection of bilirubin

Bilirubin was dissolved at a concentration of 3 mg/mL in 0.1% PBS, containing 0.2% BSA to avoid autoxidation and 0.1% NaOH to sustain the pH to 8.0-8.4. All the processes of preparation were required to be kept away from the light and to be controlled in a few minutes. The rats were divided into control groups and BE 0.5-, 4-, 24-hr groups, respectively (n = 5-8 for each group). The procedures on the BE model rats were carried out according to the operating procedure described previously [36]. In brief, under 10% chloral hydrate induced anesthesia (3 mL kg^-1^), the left jugular vein was isolated and injected of bilirubin at a does of 17 μL/g in 5 min by intubation. The control rats underwent an identical surgical intervention but injected with the equal dose saline.

### Preparation of brain extracts

The rats were sacrificed by decapitation, and specimens of bilateral hippocampus, occipital cortex and whole cerebellum were dissected immediately, snap-frozen in liquid nitrogen and stored at −80°C until use. The preparation of the brain extracts was based on the previous reference [15]. The frozen tissue was weighed and ground using an electric homogenizer with ice-cold methanol (4 mL/g) and distilled water (0.85 mL/g) at 4°C and the mixture was vortexed. Chloroform (2 mL/g) and distilled water (2 mL/g) was added and mixed again. After keeping on ice for 15 min, the homogenate was centrifuged at 1,000 g for 15 min at 4°C. The supernatant was extracted and lyophilized for about 24 h. The metabolite mixture obtained was then weighed and dissolved in 0.6 mL of 99.5% D_2_O for NMR spectroscopy.

### Determination of serum and brain bilirubin concentration and histopathology

1.5 mL blood samples were obtained and the serum samples were separated after centrifugation at 3000 g for 15 min at 4°C. Serum bilirubin concentrations were measured by vanadate oxidation method using automatic biochemical analyzer (Mindray S-300), which utilizes the reaction of bilirubin with vanadic acid resulting in a decline of absorbance measured at 450 nm, proportionate to the bilirubin present in the sample. After weighing, brain tissues with glacial acetic acid (9.0 mL/g) were homogenized at 4°C, acetone (10 mL/g) was added and mixed again. The supernatant was extracted and used to evaluate the level of brain bilirubin concentration by an automatic biochemical analyzer. Values were expressed as the mean ± SD. Some rats in each group were sacrificed by decapitation and the brains were fixed in 4% paraformaldehyde for observing histopathological changes. Pathological sections were performed by dyeing with Mayer’s hematoxylin and eosin for light microscopic observation.

### ^1^H NMR experiments

All ^1^H NMR experiments were carried out on a Bruker AVANCE III 600 MHz NMR spectrometer, with a spectral width of 12,000 Hz. The acquisition time was 2.65 s per scan, and an additional 10-s relaxation delay was used to ensure full relaxation. The number of scans was 256. The spectra were zero-filled to 64 K, and an exponential line-broadening function of 0.3 Hz was applied to the free induction decay prior to Fourier transformation. All spectra were corrected manually for phase and baseline and referenced to the chemical shift of the methyl peak of lactate (CH_3_, 1.33 ppm) using Topspin (v2.1 pl4, Bruker Biospin, Germany).

### Data processing of NMR spectra and multivariate pattern recognition

In order to exploit all the metabolic information embedded in the spectra, all NMR spectra (−0.2 to 8.8 ppm) were then divided into integral regions with equal width of 0.01 and 0.0015 ppm (2.4 Hz) using the AMIX package. Each segment consisted of the integral of the associated NMR region except δ5.85–4.60 (containing the residual peak from the suppressed water resonance), which was set to the zero integral in the analysis. The remaining spectral segments for each NMR spectrum were normalized to the total sum of the spectral intensity to partially compensate for differences in concentration of the many metabolites in the samples. Subsequently, the normalized integral values were entered into SIMCA-P + 12.0 software (Umetrics, Umeå, Sweden) as variables and were mean-centered for multivariate data analysis. The projection to latent structure discriminant analysis (PLS-DA), which is a supervised method, was carried out for class discrimination and biomarker identification [43]. Data were visualized with a principal component (PC) scores plot of the first two principal components (PC1 and PC2) to provide the most efficient 2-D representation of the information contained [44], where each point represents an individual spectrum of a sample. The corresponding loading plot, where differential metabolite peaks were shown as positive and negative signals to indicate the relative changes of metabolites, were used to identify which spectral variables contributed to the separation of the samples on the scores plot [8,44,45].

### Target metabolic changes in brain tissues and statistical analysis

Using trimethylsilyl-propionic-2,2,3,3d_4_-acid (TSP) as the internal reference, the metabolite concentrations were determined from the spectra and normalized to the weight of the freeze-dried metabolite mixture. The concentrations of metabolites were shown in the unit of mmol/kg wet tissue weight. The statistical significance of the differences observed between the averages of each group was performed using the *t* test as implemented in the software of SPSS for Windows (version 13.0, SPSS Inc, USA). A calculated P value of <0.05 was considered to be statistically significant.

## Abbreviations

Ala: Alanine
Asp: Aspartate
BE: Bilirubin encephalopathy
CNS: Central nervous system
NMR: Nuclear magnetic resonance
Glu: Glutamate
Gln: Glutamine
GABA: γ-aminobutyric acid
Lac: Lactate
NAA: N-acetyl aspartate
Tau: Taurine
TCA: Tricarboxylic acid
UCB: Unconjugated bilirubin

## References

[1] Gourley GR (1997). Bilirubin metabolism and kernicterus. Adv Pediatr.

[2] Kaplan M, Hammerman C (2005). Understanding severe hyperbilirubinemia and preventing kernicterus: adjuncts in the interpretation of neonatal serum bilirubin. Clin Chim Acta.

[3] Maisels MJ, Baltz RD, Bhutani VK, Newman TB, Palmer H, Rosenfeld W, Stevenson DK, Weinblatt HB, Hyperbilirubinemia S (2004). Management of hyperbilirubinemia in the newborn infant 35 or more weeks of gestation. Pediatrics.

[4] Amit Y, Poznansky MJ, Schiff D (1992). Neonatal jaundice and bilirubin encephalopathy: a clinical and experimental reappraisal. Isr J Med Sci.

[5] Zelenka J, Lenicek M, Muchova L, Jirsa M, Kudla M, Balaz P, Zadinova M, Ostrow JD, Wong RJ, Vitek L (2008). Highly sensitive method for quantitative determination of bilirubin in biological fluids and tissues. J Chromatogr B.

[6] Brito MA, Rosa AI, Falcao AS, Fernandes A, Silva RF, Butterfield DA, Brites D (2008). Unconjugated bilirubin differentially affects the redox status of neuronal and astroglial cells. Neurobiol Dis.

[7] Vaz AR, Delgado-Esteban M, Brito MA, Bolanos JP, Brites D, Almeida A (2010). Bilirubin selectively inhibits cytochrome c oxidase activity and induces apoptosis in immature cortical neurons: assessment of the protective effects of glycoursodeoxycholic acid. J Neurochem.

[8] Vaz AR, Silva SL, Barateiro A, Falcao AS, Fernandes A, Brito MA, Brites D (2011). Selective vulnerability of rat brain regions to unconjugated bilirubin. Mol Cell Neurosci.

[9] Fernandes A, Falcao AS, Abranches E, Bekman E, Henrique D, Lanier LM, Brites D (2009). Bilirubin as a determinant for altered neurogenesis, neuritogenesis, and synaptogenesis. Dev Neurobiol.

[10] Sanacora G, Gueorguieva R, Epperson CN, Wu YT, Appel M, Rothman DL, Krystal JH, Mason GF (2004). Subtype-specific alterations of gamma-aminobutyric acid and glutamate in patients with major depression. Arch Gen Psychiatry.

[11] Kahn RA, Panah M, Kiffel S, Weinberger J (1997). Modulation of ischemic excitatory neurotransmitter and gamma-aminobutyric acid release during global temporary cerebral ischemia by local nitric oxide synthase inhibition. Anesth Analg.

[12] Campos F, Perez-Mato M, Agulla J, Blanco M, Barral D, Almeida A, Brea D, Waeber C, Castillo J, Ramos-Cabrer P (2012). Glutamate excitoxicity is the key molecular mechanism which is influenced by body temperature during the acute phase of brain stroke. Plos one.

[13] Wang X, Li YH, Li MH, Lu J, Zhao JG, Sun XJ, Zhang B, Ye JL (2012). Glutamate level detection by magnetic resonance spectroscopy in patients with post-stroke depression. Eur Arch Psy Clin N.

[14] Gao HC, Zhu H, Song CY, Lin L, Xiang Y, Yan ZH, Bai GH, Ye FQ, Li XK (2013). Metabolic changes detected by ex vivo high resolution ^1^H NMR spectroscopy in the striatum of 6-OHDA-induced Parkinson's rat. Mol Neurobiol.

[15] Gao H, Xiang Y, Sun N, Zhu H, Wang Y, Liu M, Ma Y, Lei H (2007). Metabolic changes in rat prefrontal cortex and hippocampus induced by chronic morphine treatment studied ex vivo by high resolution ^1^H NMR spectroscopy. Neurochem Int.

[16] Mangia S, Tkac I, Gruetter R, Van de Moortele PF, Maraviglia B, Ugurbil K (2007). Sustained neuronal activation raises oxidative metabolism to a new steady-state level: evidence from ^1^H NMR spectroscopy in the human visual cortex. J Cereb Blood Flow Metab.

[17] Mangia S, Giove F, Tkac I, Logothetis NK, Henry PG, Olman CA, Maraviglia B, Di Salle F, Ugurbil K (2009). Metabolic and hemodynamic events after changes in neuronal activity: current hypotheses, theoretical predictions and in vivo NMR experimental findings. J Cereb Blood Flow Metab.

[18] Liu K, Ye XJ, Hu WY, Zhang GY, Bai GH, Zhao LC, He JW, Zhu H, Shao JB, Yan ZH, Gao HC (2013). Neurochemical changes in the rat occipital cortex and hippocampus after repetitive and profound hypoglycemia during the neonatal period: an ex vivo (1)H magnetic resonance spectroscopy study. Mol Neurobiol.

[19] Globus MY, Busto R, Dietrich WD, Martinez E, Valdes I, Ginsberg MD (1988). Effect of ischemia on the in vivo release of striatal dopamine, glutamate, and gamma-aminobutyric acid studied by intracerebral microdialysis. J Neurochem.

[20] Fernandes A, Silva RF, Falcao AS, Brito MA, Brites D (2004). Cytokine production, glutamate release and cell death in rat cultured astrocytes treated with unconjugated bilirubin and LPS. J Neuroimmunol.

[21] Falcao AS, Fernandes A, Brito MA, Silva RF, Brites D (2005). Bilirubin-induced inflammatory response, glutamate release, and cell death in rat cortical astrocytes are enhanced in younger cells. Neurobiol Dis.

[22] Chassain C, Bielicki G, Donnat JP, Renou JP, Eschalier A, Durif F (2005). Cerebral glutamate metabolism in Parkinson's disease: an in vivo dynamic (13)C NMS study in the rat. Exp Neurol.

[23] Haberg A, Qu H, Haraldseth O, Unsgard G, Sonnewald U (1998). In vivo injection of [1-^13^C]glucose and [1,2-^13^C]acetate combined with ex vivo ^13^C nuclear magnetic resonance spectroscopy: a novel approach to the study of middle cerebral artery occlusion in the rat. J Cerebr Blood F Met.

[24] Chih CP, Roberts EL (2003). Energy substrates for neurons during neural activity: a critical review of the astrocyte-neuron lactate shuttle hypothesis. J Cereb Blood Flow Metab.

[25] Katoh-Semba R (1976). Studies on cellular toxicity of bilirubin: effect on brain glycolysis in the young rat. Brain Res.

[26] Noir BA, Boveris A, Garaza Pereira AM, Stoppani AO (1972). Bilirubin: a multi-site inhibitor of mitochondrial respiration. FEBS Lett.

[27] Ollinger R, Bilban M, Erat A, Froio A, McDaid J, Tyagi S, Csizmadia E, Graca-Souza AV, Liloia A, Soares MP, Otterbein LE, Usheva A, Yamashita K, Bach FH (2005). Bilirubin: a natural inhibitor of vascular smooth muscle cell proliferation. Circulation.

[28] Younes RN, Poggetti RS, Fontes B, Itinoshe MM, Yoshida VM, Carvalho ME, Birolini D (2007). Obstructive jaundice induces early depression of mitochondrial respiration in rat hepatocytes. Acta Cir Bras.

[29] Ogasawara N, Watanabe T, Goto H (1973). Bilirubin: a potent inhibitor of NAD^+^-linked isocitrate dehydrogenase. Biochim Biophys Acta.

[30] Isaacks RE, Bender AS, Kim CY, Prieto NM, Norenberg MD (1994). Osmotic regulation of myo-inositol uptake in primary astrocyte cultures. Neurochem Res.

[31] Saransaari P, Oja SS (2000). Taurine and neural cell damage. Amino Acids.

[32] Zhang X, Liu H, Wu J, Zhang X, Liu M, Wang Y (2009). Metabonomic alterations in hippocampus, temporal and prefrontal cortex with age in rats. Neurochem Int.

[33] Watanabe H, Fukatsu H, Katsuno M, Sugiura M, Hamada K, Okada Y, Hirayama M, Ishigaki T, Sobue G (2004). Multiple regional ^1^H-MR spectroscopy in multiple system atrophy: NAA/Cr reduction in pontine base as a valuable diagnostic marker. J Neurol Neurosurg Psychiatry.

[34] Wang X, Wu W, Hou BL, Zhang P, Chineah A, Liu F, Liao W (2008). Studying neonatal bilirubin encephalopathy with conventional MRI, MRS, and DWI. Neuroradiology.

[35] Roger C, Koziel V, Vert P, Nehlig A (1993). Effects of bilirubin infusion on local cerebral glucose utilization in the immature rat. Brain Res Dev Brain Res.

[36] Roger C, Koziel V, Vert P, Nehlig A (1995). Regional cerebral metabolic consequences of bilirubin in rat depend upon post-gestational age at the time of hyperbilirubinemia. Dev Brain Res.

[37] Falcao AS, Silva RF, Pancadas S, Fernandes A, Brito MA, Brites D (2007). Apoptosis and impairment of neurite network by short exposure of immature rat cortical neurons to unconjugated bilirubin increase with cell differentiation and are additionally enhanced by an inflammatory stimulus. J Neurosci Res.

[38] Hanko E, Hansen TW, Almaas R, Rootwelt T (2006). Recovery after short-term bilirubin exposure in human NT2-N neurons. Brain Res.

[39] Hansen TW, Cashore WJ, Oh W (1992). Changes in piglet auditory brainstem response amplitudes without increases in serum or cerebrospinal fluid neuron-specific enolase. Pediatr Res.

[40] Hansen TW, Nietsch L, Norman E, Bjerre JV, Hascoet JM, Mreihil K, Ebbesen F (2009). Reversibility of acute intermediate phase bilirubin encephalopathy. Acta Paediatr.

[41] Prasanthi RP, Devi CB, Basha DC, Reddy NS, Reddy GR (2010). Calcium and zinc supplementation protects lead (Pb)-induced perturbations in antioxidant enzymes and lipid peroxidation in developing mouse brain. Int J Dev Neurosci.

[42] Guzzetta F, Deodato F, Rando T (2000). Brain ischemic lesions of the newborn. Childs Nerv Syst.

[43] Coen M, Lenz EM, Nicholson JK, Wilson ID, Pognan F, Lindon JC (2003). An integrated metabonomic investigation of acetaminophen toxicity in the mouse using NMR spectroscopy. Chem Res Toxicol.

[44] Gao H, Dong B, Liu X, Xuan H, Huang Y, Lin D (2008). Metabonomic profiling of renal cell carcinoma: high-resolution proton nuclear magnetic resonance spectroscopy of human serum with multivariate data analysis. Anal Chim Acta.

[45] Gao H, Lu Q, Liu X, Cong H, Zhao L, Wang H, Lin D (2009). Application of ^1^H NMR-based metabonomics in the study of metabolic profiling of human hepatocellular carcinoma and liver cirrhosis. Cancer Sci.

